# New Frontiers in Pancreatic Cancer Management: Current Treatment Options and the Emerging Role of Neoadjuvant Therapy

**DOI:** 10.3390/medicina60071070

**Published:** 2024-06-28

**Authors:** Sofia Dallavalle, Gabriele Campagnoli, Paola Pastena, Alessandro Martinino, Davide Schiliró, Francesco Giovinazzo

**Affiliations:** 1 Faculty of Medicine and Surgery, University of Milan, 20122 Milan, Italy; sofia.dallavalle@studenti.unimi.it (S.D.); gabriele.campagnoli@studenti.unimi.it (G.C.); 2 Department of Medicine, Stony Brook Medicine, Stony Brook, NY 11794, USA; paolapastena@outlook.com; 3 Department of Surgery, Duke University, Durham, NC 27710, USA; 4 Department of Surgery, Saint Camillus Hospital, 31100 Treviso, Italy; 5 Department of Surgery, UniCamillus-Saint Camillus International University of Health Sciences, 00131 Rome, Italy; 6 Department of Surgery, Agostino Gemelli University Hospital, 00168 Rome, Italy

**Keywords:** pancreatic cancer, pancreatic ductal adenocarcinoma, management, resectability, neoadjuvant therapy

## Abstract

Pancreatic ductal adenocarcinoma (PDAC) ranks among the 15 most prevalent cancers globally, characterized by aggressive growth and late-stage diagnosis. Advances in imaging and surgical techniques have redefined the classification of pancreatic PDAC into resectable, borderline resectable, and locally advanced pancreatic cancer. While surgery remains the most effective treatment, only 20% of patients are eligible at diagnosis, necessitating innovative strategies to improve outcomes. Therefore, traditional treatment paradigms, primarily surgical resection for eligible patients, are increasingly supplemented by neoadjuvant therapies (NAT), which include chemotherapy, radiotherapy, or a combination of both. By administering systemic therapy prior to surgery, NAT aims to reduce tumor size and increase the feasibility of complete surgical resection, thus enhancing overall survival rates and potentially allowing more patients to undergo curative surgeries. Recent advances in treatment protocols, such as FOLFIRINOX and gemcitabine-nab-paclitaxel, now integral to NAT strategies, have shown promising results in increasing the proportion of patients eligible for surgery by effectively reducing tumor size and addressing micrometastatic disease. Additionally, they offer improved response rates and survival benefits compared to traditional regimes. Despite these advancements, the role of NAT continues to evolve, necessitating ongoing research to optimize treatment regimens, minimize adverse effects, and identify patient populations that would benefit most from these approaches. Through a detailed analysis of current literature and recent clinical trials, this review highlights the transformative potential of NAT in managing PDAC, especially in patients with borderline resectable or locally advanced stages, promising a shift towards more personalized and effective management strategies for PDAC.

## 1. Introduction

Pancreatic cancer ranks among the 15 most common malignant tumors worldwide and primarily presents as pancreatic ductal adenocarcinoma (PDAC) [1,2]. This neoplasm is characterized by rapid growth, strong invasiveness, and a high degree of malignancy, with most of the patients getting diagnosed when the cancer is already in the advanced stage [1,3].

Advancements in pancreatic imaging and surgical methods have identified a new subgroup of pancreatic tumors known as borderline resectable cancers, leading to a revised classification of pancreatic cancer into resectable (R), borderline resectable (BR), and locally advanced (LA) PDAC [4,5,6]. 

Surgery offers the best chance of survival. However, only 20% of the patients are suitable for resection at diagnosis [6,7]. Depending on tumor localization, different surgical approaches are adopted in patients with resectable pancreatic cancer [8,9]. Recent advances in minimally invasive procedures, including laparoscopic and robotic techniques, have reduced perioperative complications [10,11]. Additionally, the development of local ablative techniques in recent years offers an alternative to traditional surgical intervention [10,12,13,14].

Chemotherapy and radiotherapy are key approaches for managing borderline resectable disease and locally advanced disease. The treatments can be palliative, adjuvant (administered post-surgical resection), or neoadjuvant (administered to the patient in order to render the lesion suitable for resection) [15,16,17,18]. The main chemotherapeutic protocols currently used are gemcitabine-nab-paclitaxel, FOLFIRINOX, and nal-irinotecan [15,19]. In the realm of radiotherapy, stereotactic body radiation therapy represents a cutting-edge frontier [20].

Although advancements in surgical techniques and adjuvant chemotherapy have enhanced treatment outcomes for resectable and borderline resectable PDAC, achieving long-term survival remains a challenge for most patients [9]. In fact, post-operative complications and patient deterioration can hinder the completion of adjuvant chemotherapy regimens [21]. Neoadjuvant therapy (NAT) offers a potential paradigm shift by administering systemic therapy, such as chemotherapy or chemoradiotherapy, before surgery. This approach holds promise for addressing the limitations of traditional treatment by downsizing tumors, potentially increasing the number of patients eligible for curative surgery [8], improving the chances of achieving complete tumor removal with clear margins [21], and allowing for targeting micrometastases present at diagnosis, potentially improving long-term survival [9]. Identifying the most effective neoadjuvant regimens, including chemotherapy alone or combined with radiotherapy, requires further investigation [22]. This review delves into the latest research protocols employed in the investigation of neoadjuvant therapy for pancreatic ductal adenocarcinoma, analyzing the benefits and ongoing challenges aimed at refining treatment strategies.

## 2. Epidemiology and Prognosis of Pancreatic Cancer

Pancreatic cancer is a very frequent malignant tumor commonly presenting as pancreatic adenocarcinoma (PDAC) [1]. Because of its rapid growth, strong invasiveness, and high degree of malignancy, it is characterized by poor prognosis. Most of the patients receive diagnosed when the cancer is already in an advanced stage, with a low resection rate and poor treatment effects [1,3].

Modifiable risk factors of pancreatic cancer include cigarette smoking (1.7-fold increased risk compared to non-smokers), obesity (1.6-fold increased risk compared to non-obese people), alcohol intake (1.6-fold increased risk for those consuming >6 drinks per day compared to those consuming one drink per day), diabetes (1.5-fold increased risk compared to non-diabetic individuals), and pancreatitis (2–3-fold increased risk for patients with chronic pancreatitis). Genetic predispositions also play a significant role in increasing the risk of pancreatic cancer. Notably, variants in the genes *BRCA2, BRCA1, ATM, CDKN2A,* and *P16* are commonly observed in patients diagnosed with this disease [23]. Observational studies consistently demonstrated an elevated risk of pancreatic cancer in individuals with a first-degree relative diagnosed with the disease [23]. Prospective studies further solidified this association, revealing a 6.79-fold (95% CI: 4.54–9.75) increased risk for first-degree relatives in familial pancreatic cancer kindreds (families with at least two affected members) [23]. Unlike some cancers where family history correlates with a younger age of onset, pancreatic cancer displays minimal age difference (maximum 6 years) between those with and without a family history [23]. Additionally, pancreatic cancer exhibits familial clustering with other malignancies. Relatives of pancreatic cancer patients have shown increased risks for breast, ovarian, prostate, colon, bile duct, and liver cancers [23]. Klein et al. (2021) reported large-scale histopathological analyses revealing a striking similarity between familial and sporadic pancreatic cancers, with no statistically significant differences detected. This observation extends to the somatic mutational profile, as familial and sporadic pancreatic cancers exhibit comparable genetic alterations. Despite this similarity in the tumors themselves, individuals with a family history display a higher prevalence of precancerous lesions in the surrounding healthy pancreatic tissue compared to those without a family history. These findings suggest a highly similar underlying genetic basis for both familial and sporadic pancreatic cancers. However, individuals with a family history appear to be predisposed to developing precursor lesions, some of which may progress to invasive cancer [23]. Over the past decade, significant strides have been made in elucidating the genetic underpinnings of familial pancreatic cancer. Genome-wide association studies and targeted sequencing efforts have uncovered an array of both rare, high-penetrance, and common, low-penetrance genetic variants associated with an increased risk of developing pancreatic cancer. Despite these remarkable advances, the identified genetic alterations account for only a fraction of the estimated heritability (20–25%), highlighting the substantial genetic complexity underlying this disease [23]. Klein et al. highlight the prevalence of BRCA2 mutations in patients with pancreatic cancer ranging from 1.4% to 16%, with the higher rates observed in individuals with a family history of pancreatic, ovarian, or breast cancer. Indeed, carriers of BRCA2 mutations exhibit a 3.5–5.8-fold increased risk of developing pancreatic cancer compared to the general population, and mutations in the BRCA1 gene, encoding a crucial protein involved in DNA repair, are also associated with an elevated risk of pancreatic cancer. While the risk associated with BRCA1 mutations is generally lower than that of BRCA2 mutations, carriers in this case still face a 2.7–4.1-fold increased risk [23]. According to Klein’s analysis, pathogenic variants in the CDKN2A gene, initially linked to melanoma susceptibility, have also been implicated in pancreatic cancer risk, and carriers of CDKN2A mutations exhibit a substantial 12–38-fold increased risk of developing pancreatic cancer, with a prevalence of up to 2.5% in familial cases. Among individuals with a specific CDKN2A mutation undergoing early detection screening, 7.3% were found to harbor pancreatic cancer [23]. The identification of these high-penetrance gene mutations has profound implications for both patients and their at-risk relatives [23]. Additionally, genetic testing can inform early detection strategies for at-risk relatives, enabling proactive surveillance and potentially reducing pancreatic cancer mortality. Continued research efforts are essential to further unravel the genetic complexity of this disease and identify novel therapeutic targets and preventive strategies [23].

The number of PDAC cases is continuously rising globally, with no corresponding decline in mortality rates. Between 1990 and 2019, both the incidence and mortality of PDAC increased by almost 170% [3]. In 2020, the number of new cases of PDAC was 495,773, ranking it among the 15th most common malignant tumors worldwide, and the estimated number of deaths due to PDAC for the same year was 466,003, placing it seventh among cancer deaths [1,2]. The mortality rate is the highest in Europe (7.2 deaths per 100,000 people), followed by North America (6.5 per 100,000 people), while it is the lowest in East Africa (1.2 per 100,000 people). The 1-year overall survival (OS) rate of patients with PDAC is very low (24%), while the 5-year OS is around 9% [2]. In the US, approximately 50% of the patients have distant-stage PDAC, with a 5-year survival rate of 2.9%, while 11% of patients have localized-stage PDAC and a 5-year survival rate of 39.4% [1]. Tumor characteristics (staging, size, invasion site), patient status, and treatment represent the main factors influencing PDAC prognosis [1].

## 3. Staging of Pancreatic Cancer

The primary goal in managing suspected or confirmed pancreatic cancer is to assess the potential for surgical resection, as surgery offers the best chance of survival. However, due to locally advanced or metastatic disease, fewer than 20% of patients are typically eligible for surgery at the time of diagnosis [6,7]. Therefore, identifying patients who are likely to benefit from surgery remains crucial to maximize treatment effectiveness and prevent unnecessary procedures in those with unresectable diseases. In fact, in cases where surgery is not feasible, attempting resection could lead to significant morbidity and mortality [6]. 

Advancements in pancreatic imaging and surgical methods have led to the recognition of a unique subgroup of pancreatic tumors, blurring the distinction between resectable and locally advanced unresectable disease: borderline resectable cancers [4]. This subgroup is defined according to three dimensions: anatomical, biological, and conditional [5,8]. The anatomic definition is based on tumor proximity or invasion of the superior mesenteric vein or portal vein, tumor contact with the superior mesenteric artery and/or celiac artery, or tumor proximity to the common hepatic artery. The biological definition relies on a serum CA 19–9 level exceeding 500 IU/mL and/or the presence of positive regional lymph node metastases confirmed by biopsy or PET-CT. Lastly, the conditional definition is based on the patient’s surgical risk due to host-related factors, such as performance status and comorbidities [5].

There is now consensus that categorizing pancreatic ductal adenocarcinoma (PDAC) into resectable (R), borderline resectable (BR), and locally advanced (LA) significantly aids in treatment planning and patient management [5]. However, different guidelines often exhibit ambiguity due to the unclear distinction between clearly resectable situations and borderline resectable cases [7], and disagreements regarding the selection of a potentially curative or palliative treatment strategy in borderline resectable cases are concerning, as the chosen treatment significantly impacts prognosis [6]. Therefore, accurate reporting of the staging criteria used and their rigorous application as prerequisites for enrollment remain crucial [9]. In practice, surgical resectability is determined by various empirical sets of staging criteria. These criteria assess the anatomy of the primary tumor and its relationships with the superior mesenteric vein and portal vein, superior mesenteric artery (SMA), common hepatic artery and its branches, and the coeliac trunk [9]. The most pertinent classification criteria are those established by the Americas Hepato-Pancreato-Biliary Association, the Society of Surgical Oncology, the Society for Surgery of the Alimentary Tract (AHPBA/SSO/SSAT), the University of Texas MD Anderson Cancer Center, the Alliance for Clinical Trials in Oncology, and the NCCN [9]. According to the NCCN staging criteria, pancreatic cancer can be defined as: resectable when there is no tumor contact, or contact with less than 180° without vein contour irregularity in relation with superior mesenteric vein or portal vein, and when there is no arterial contact in relation with superior mesenteric artery, celiac artery, or central hepatic artery; borderline resectable when there is a solid tumor with a contact of more than 180°, or less than 180° and irregularity of the vein or thrombosis of the vein but with vessel (suitable for safe resection and vein reconstruction) in relation with SMV/PV or solid tumor contact with inferior vena cava, or when there is contact of less of 180° with CA, contact of 180° or more with CA without the involvement of aorta and intact and uninvolved gastroduodenal artery, contact with CHA without extension to CA/hepatic artery bifurcation (suitable for safe resection and vein reconstruction); unresectable locally advanced when there is unreconstructable involvement of SMV/PV, contact with SMV, more than 180° involvement of SMA or CA, or contact with CA and aorta; unresectable metastatic when there are distant metastases, including non-regional lymph nodes [5]. Beyond resectability staging criteria, pancreatic cancer staging also follows cancer TNM staging from stage I to stage IV, established by the American Joint Committee on Cancer [24].

**Figure 1:**
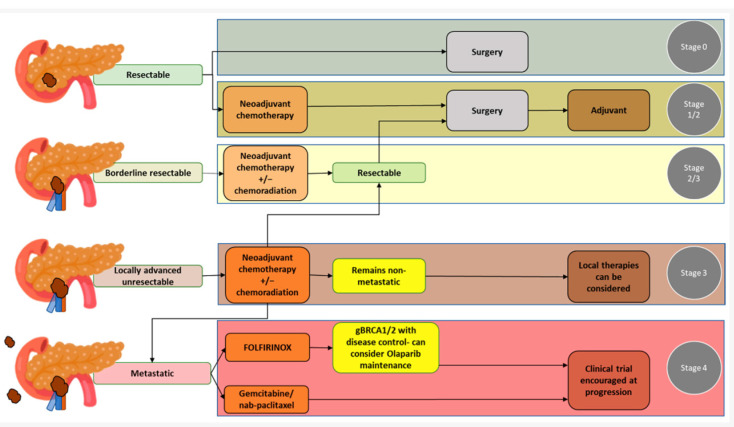
Pancreatic adenocarcinoma classification according to the resectability status [25].

The classification of pancreatic cancer according to the resectability status is reported in Figure 1, together with the main treatment options.

## 4. Current Treatment Strategies

### 4.1. Overview

Following the completion of staging protocols and discussions in a multidisciplinary tumor board, a treatment decision congruent with the findings should be taken [8].

As a general principle, the primary approach for potentially curative intervention in pancreatic cancer is surgical resection of the primary tumor. After radiographic assessment, only individuals showing a high likelihood of complete tumor removal without margin involvement are deemed suitable candidates for primary surgical intervention, but they constitute less than 20% of diagnosed cases. Additionally, it is crucial to consider that in cases with severe comorbidities or profound malnutrition, despite optimal supportive care, surgery might be avoided even if technically feasible, requiring other treatment strategies [8,9].

In individuals diagnosed with resectable pancreatic cancer who are eligible for adjuvant chemotherapy, the five-year overall survival rate ranges approximately between 30–50%. For those presenting with borderline-resectable cancer, a brief course of neoadjuvant therapy preceding surgical intervention becomes necessary to enhance resectability, resulting in a one-year overall survival rate of about 75%. Additionally, specific patients initially presenting with locally advanced, unresectable, non-metastatic pancreatic cancer may become candidates for surgical resection following induction chemotherapy (with or without radiotherapy), demonstrating improved overall survival compared to those not undergoing resection [9].

**Figure 2:**
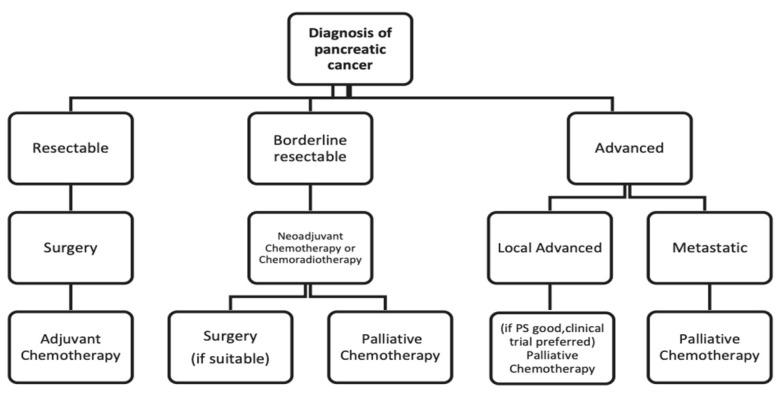
Treatment algorithm for pancreatic cancer [26].

The algorithm to be used when evaluating which strategy to adopt in order to treat a patient with pancreatic cancer can be summarized in Figure 2.

### 4.2. Surgical Resection

The choice of surgical approach for resection is dictated by the size and location of the tumor. Patients with tumors situated in the pancreatic head typically undergo pancreatoduodenectomy, commonly known as the Whipple procedure. Conversely, patients with tumors in the body or tail of the pancreas typically undergo distal pancreatectomy, entailing resection of the pancreatic body and tail, along with splenectomy [8]. An additional crucial consideration is the presence of lymph node involvement in patients diagnosed with resectable pancreatic ductal adenocarcinoma, which serves as a significant prognostic indicator. The survival advantage conferred by extended lymphadenectomy during pancreatectomy is subject to debate when compared to standard lymphadenectomy [27].

To achieve optimal medical clearance and enhance the R0 resection rate, it is advised to perform a dissection of the right hemicircumference of the superior mesenteric artery to the right of the celiac trunk. In instances of venous involvement, the option of complete venous resection of the portal vein or superior mesenteric vein, followed by vessel reconstruction to obtain R0 status, is possible but associated with diminished survival outcomes [8].

The radical antegrade modular pancreatosplenectomy procedure represents a modification of the standard distal pancreatectomy, designed to extend the operative approach utilized for pancreatic head cancers to encompass cancers affecting the body and tail of the pancreas, particularly focusing on lymph node dissection and tangential margins [28]. Additionally, radical anterograde modular pancreatosplenectomy with dissection of the left hemicircumference of the superior mesenteric artery to the left of the celiac trunk ensures R0 resection [8].

Over the past decade, significant progress in the surgical management of pancreatic cancer has enhanced outcomes for patients with non-metastatic, operable pancreatic cancer. Notably, this includes advancements in minimally invasive procedures, such as laparoscopic and robotic techniques, which have markedly improved surgical results. Two significant developments in the field include minimally invasive distal pancreatectomy and minimally invasive pancreatoduodenectomy [10,11].

Minimally invasive pancreatectomy has led to improved perioperative outcomes without compromising oncologic results [10]. In fact, it has been shown to diminish post-operative complications compared to open distal pancreatectomy and, under the guidance of proficient surgeons, can be regarded as the preferred approach for distal pancreatic cancer. [10,29]. Specifically, it has been associated with less operative blood loss, reduced delayed gastric emptying, shorter time to functional recovery, and improved quality of life scores [10].

Vascular involvement poses a significant challenge in minimally invasive pancreaticoduodenectomy. However, robotic pancreatoduodenectomy has proven to be a safe and effective alternative to traditional open pancreatoduodenectomy when performed by skilled surgeons [30]. Similarly, total laparoscopic pancreatoduodenectomy, even with major vascular resection, is a viable option that offers safety and feasibility comparable to open procedures, with similar morbidity, mortality, and oncological outcomes [11].

### 4.3. Ablative Techniques

For patients who remain unresectable even after neoadjuvant therapy (only 25% of the patients can be converted to resection), a solution can be represented by locoregional ablative techniques [10,12]. The three main ablative techniques are radiofrequency ablation (RFA), irreversible electroporation (IRE), and high-intensity focused ultrasounds (HIFU) [10,12,13,14].

IRE involves the insertion of needle electrodes into and around the periphery of pancreatic duct adenocarcinoma. The ablative effect of this non-thermal technique relies on the delivery of high-voltage electrical pulses from the electrodes, which disrupt the permeability of the plasma membrane. This disruption creates nanopores, disturbs cellular homeostasis, and ultimately induces apoptosis [10,12]. With IRE, the connective tissue matrix supposedly remains unaffected, which may lead to the preservation of vascular and ductal structures [12]. Additionally, this technique could provide a potential bridge from locally advanced to resectable tumors and may increase survival by several months [10].

RFA involves the insertion of needles into the center of the tumor. This procedure operates on the principle of heat generation through the application of a high-frequency alternating current. The resulting thermal coagulation and protein denaturation lead to the destruction of the tumor [12].

HIFU utilizes therapeutic ultrasound waves at a frequency of 0.8–1.0 MHz, emitted by a transducer with a focal length of 15 cm and a diameter of 20 cm. Performed under general anesthesia, this technique induces cell destruction and tissue necrosis primarily through thermal and mechanical effects. Notably, it does not impact vessels larger than 200 µm in diameter, making it particularly valuable for patients with cancers near large vessels. Post-ablation imaging, such as CT or MRI, shows a non-perfused volume in each treated lesion, and patients have reported significant pain relief. This suggests that HIFU is effective for both pain management and inhibition of tumor progression in advanced cases [13].

Overall, studies on RFA, IRE, and HIFU suggest that these ablative therapies are relatively safe and could contribute to a temporary improvement in the local control of unresectable pancreatic adenocarcinoma. However, these techniques are not widely available to patients and are often applied within research protocols and clinical trials due to their experimental nature and need for specialized equipment and expertise [14]. Major limitations in the use of IRE in PDAC treatment include its high risk due to vicinity with vital structures, challenges in precise electrode placement, post-treatment imaging acquisition due to reactive edema, and complication rates [31]. The limitations of RFA use are more related to the variability of clinical outcomes, the fact that there is limited long-term data, and the presence of technical difficulties in needle placement [32]. HIFU limitations in treating PDAC include possibly short-duration pain relief and the potential for minor adverse effects, such as skin burns [13]. More studies, data collection, and surgeon training are needed before seeing RFA, IRE, and HIFU getting routinely involved in the treatment of pancreatic cancer [14].

### 4.4. Chemotherapy

Currently, chemotherapy is primarily used in two scenarios: as palliative care for patients with locally advanced or metastatic disease and as adjuvant therapy following surgery [15,16]. Additionally, recent research is considering chemotherapy as a neoadjuvant strategy to allow resection in initially non-suitable patients [15].

Patients with locally advanced or metastatic PDAC are usually approached with palliative intent [16]. Starting from 1997, several trials have been run in order to evaluate the overall survival rate, progression-free survival rate, and possible toxicity of different chemotherapeutic agents [15]. Two therapies have emerged as the standard of care for first-line treatment, gemcitabine-nab-paclitaxel and FOLFIRINOX (5-fluorouracil, folinic acid, irinotecan, and oxaliplatin), and can be offered to patients with good performance status. However, these therapies have limited effectiveness, and more effective pharmacotherapeutic options are needed [15,19]. Additionally, compared to other cancers, the survival benefits for metastatic cancer remain poor even with intensive combination chemotherapy, highlighting the urgent need for further research to develop new strategies [15].

Chemotherapy is not only relevant as a palliative treatment for metastatic patients but also for patients with resectable disease after surgery. Indeed, 90% of the patients relapse after surgery, and adjuvant strategies can be used to increase the fraction of patients with long-term survival [15]. In fact, adjuvant chemotherapy after surgery in PDAC is evolving. The standard approach is 5FU monotherapy or gemcitabine, but two recent trials, ESPAC4 and PRODIGE24, have shown better outcomes [16].

The main trials of the different chemotherapeutic agents are summarized in Table 1.

### 4.5. Radiotherapy and Chemoradiotherapy

Traditionally, conventionally fractioned radiation therapy (delivering 40–60 Gy in 1.8–2.0 Gy fractions) has been used to treat locally advanced pancreatic cancer, but its efficacy in improving survival, when compared to chemotherapy alone, has remained controversial. Randomized trials, including the LAP-07 trial, showed no significant survival benefit from adding conventional chemoradiotherapy to chemotherapy [17,18,41].

The biggest problem related to chemoradiotherapy for pancreatic cancer is that the pancreas is surrounded by radiosensitive gastrointestinal organs, such as the stomach and duodenum. The anatomical situation makes it difficult to deliver high doses to the tumor without irradiating the surrounding organs; thus, radiotherapeutic treatment could induce gastrointestinal toxicity and upper gastrointestinal bleeding [18,42].

Given the lack of clear survival benefits and the higher incidence of adverse events with chemoradiotherapy, the strategy has shifted towards starting with combination chemotherapy and considering the addition of chemoradiotherapy only if there are potential benefits [43].

In recent years, advancements in radiation delivery techniques like Stereotactic Body Radiotherapy (SBRT) have provided new options. SBRT delivers highly focused ablative doses to a limited target volume with high precision over a short period (3–5 sessions over 1–2 weeks), potentially overcoming radioresistance and allowing for higher doses without severe side effects [17,20,43]. In addition, thanks to precise radiation focus reached thanks to magnetic resonance-guided techniques, it is possible to reach higher dose escalation and convert unresectable tumors to operable cases [41]. Radiotherapy is, indeed, also used neoadjuvantly (before surgery) to control tumors and induce shrinkage, potentially converting unresectable tumors into operable ones [43].A recent study showed that chemotherapy combined with SBRT led to improved median survival (13.9 months) compared to chemotherapy combined with intensity-modulated radiotherapy (IMRT; 12.2 months), external-beam radiotherapy (EBRT; 11.6 months), and chemotherapy alone (10.2 months) [20]. Additionally, radiotherapy not only aids in pain management, but also improves the probability of local cure in the treatment of locally advanced pancreatic cancers [44]. However, SBRT comes with some limitations: despite its precision, SBRT still carries the risk of gastrointestinal toxicities due to the fact that radiosensitive organs like the stomach and duodenum are in close contact with the pancreas; moreover, even if it is true that some cases can be converted from unresectable to resectable by using SBRT as neoadjuvant therapy, the conversion rate is really low (5–18%) [20,41]. The clinical outcomes SBRT therapy has compared to other treatment strategies are such that it is gaining traction in clinical practice; however, due to the abovementioned limitations, clinical trials are run in order to better study the advantages related to SBRT as a treatment of choice and further improve its applications [17,20,43]. 

## 5. Neoadjuvant Therapy

### 5.1. Overview

Neoadjuvant therapy has emerged as a promising strategy to improve outcomes in pancreatic ductal adenocarcinoma, which is characterized by a limited window of surgical opportunity. In fact, only approximately 20% of patients are diagnosed with resectable tumors at presentation [45]. The high rate of positive surgical margins (36–64%) following resection of borderline resectable tumors, which is associated with poorer survival outcomes, has spurred research efforts investigating the use of neoadjuvant therapies [16].

In particular, this therapeutic strategy aims to expand the window of surgical resectability by downsizing tumors and enhancing operability. Consequently, a larger pool of patients with PDAC can potentially benefit from a surgical intervention [9]. By promoting tumor regression prior to surgery, NAT increases the likelihood of achieving a complete (R0) resection, a crucial factor associated with improved long-term survival in PDAC patients [16].

Therefore, in recent years, numerous clinical trials have been conducted to evaluate the efficacy of these therapies in patients with resectable, Borderline resectable, and locally advanced PDAC [16].

### 5.2. The Evolving Role of Neoadjuvant Therapy in Pancreatic Cancer

Despite the established success of neoadjuvant chemoradiotherapy (NCRT) in rectal cancer, with reported tumor downstaging rates of up to 50–60%, the application of NAT in PDAC faced initial challenges. Specifically, the majority of early chemotherapy regimens for PDAC lacked efficacy, discouraging patient participation in NAT trials. In fact, traditional regimens, such as gemcitabine monotherapy, demonstrated limited efficacy in the neoadjuvant setting, with response rates typically below 12%. Additionally, limitations in conventional imaging techniques, like CT and MRI scans, hampered the accurate assessment of tumor response to NAT, making it difficult to definitively determine resectability or pathological response [9,45,46,47,48]. Another significant therapeutic challenge was represented by its inherent chemoresistance compared to other cancer types [9].

However, the landscape of NAT in PDAC is evolving due to several advancements: more efficacious chemotherapy combinations like FOLFIRINOX and gemcitabine (GnP) are demonstrating promise in the neoadjuvant setting [45]. The National Comprehensive Cancer Network (NCCN) guidelines now endorse NAT for borderline resectable (BR) PDAC, while upfront surgery remains the preferred approach for resectable disease in most cases [45]. Moreover, encouraging results from recent trials have led to an increase in the utilization of NAT with FOLFIRINOX and GnP regimens, even for patients with initially resectable disease [45].

The traditional paradigm of upfront surgery in PDAC is undergoing revision. Growing evidence supports total neoadjuvant treatment (chemotherapy followed by chemoradiation) as a promising approach [16]. While further research is necessary, NAT is emerging as a valuable tool for improving resectability, potentially selecting patients who will derive the most benefit from surgery, and potentially identifying aggressive diseases earlier through improved tumor control [22].

### 5.3. Advantages and Benefits of Neoadjuvant Therapy

The benefits of NAT in PDAC are multifaceted. Downsizing tumors can potentially increase the likelihood of achieving complete surgical removal with clear margins (R0 resection), which is associated with improved long-term survival [16]. Beyond downstaging, the ability to assess a patient’s response to NAT can inform surgical decision-making, potentially identifying those who will benefit most from radical surgery [45]. NAT may also serve as a tool for identifying patients with a high risk of aggressive disease progression who might not benefit from surgery [16]. In fact, pancreatic surgery is a complex procedure that can lead to complications, potentially delaying or entirely preventing patients from receiving adjuvant chemotherapy after surgery. Studies report that up to 20% of patients experience delays, and 50% require reduced chemotherapy doses in the adjuvant setting and induction chemotherapy can identify patients who develop metastatic disease during treatment (around 14–36%), sparing them from the morbidity associated with unnecessary surgery [49].

The role of neoadjuvant therapy in borderline-resectable disease is also promising. Studies suggest it may improve OS without necessarily increasing resection rates [9]. Notably, for patients with initially unresectable PDAC, neoadjuvant chemotherapy combined with or without radiotherapy can convert the disease to a resectable state in approximately 20% of cases, even in the absence of a clear response on imaging studies. This approach translates to improved survival outcomes for this patient population [9].

The growing body of evidence supporting the benefits of neoadjuvant therapy, particularly for borderline-resectable and locally advanced cases, is leading to a paradigm shift in the treatment of even resectable PDAC towards a neoadjuvant approach [45].

Beyond the established advantages, neoadjuvant chemotherapy offers additional theoretical benefits compared to adjuvant therapy. It has the potential to more effectively target micrometastases present at the time of surgery by achieving greater tissue penetration due to intact tumor blood flow [16]. Additionally, studies suggest that neoadjuvant therapy may be better tolerated than adjuvant therapy in other cancers, potentially leading to higher completion rates [16].

Overall, these advantages can enhance both the duration and quality of life for many patients with pancreatic cancer, including those who are not selected for surgical removal [50].

### 5.4. Resectable Pancreatic Cancer

The definitive role of neoadjuvant therapy in resectable PDAC remains under active investigation. NAT has emerged as a promising strategy to address a crucial aspect of this therapeutic approach: enhancing resectability and potentially eradicating micrometastases through pre-operative therapy in patients with upfront resectable PDAC [40].

Recent phase II trials have not demonstrated a statistically significant improvement in OS with neoadjuvant therapies, also reporting lower completion rates for neoadjuvant chemotherapy compared to historical data, potentially due to treatment-related toxicities [51].

For instance, the SWOG 1505 trial compared perioperative mFOLFIRINOX with perioperative GEM-NabP and found no significant differences in overall survival (OS). Additionally, NORPACT-1, a multicenter, randomized phase II trial, evaluated the efficacy of neoadjuvant FOLFIRINOX chemotherapy versus upfront surgery in patients with resectable pancreatic head cancer, with the primary objective of assessing the impact of neoadjuvant therapy on overall survival compared to upfront surgery. In the neoadjuvant group, the OS was 25.1 months (95% CI: 17.2–34.9), while the upfront surgery group achieved a median OS of 38.5 months (95% CI: 27.6-NR). The 18-month OS rate was 60% (95% CI: 49–71) in the neoadjuvant arm and 73% (95% CI: 62–84) in the upfront surgery arm, aligning the survival outcomes observed in the neoadjuvant group with those reported in other neoadjuvant trials for resectable pancreatic cancer [51].

An additional limitation relates to the fact that existing neoadjuvant trials often combine patients with resectable and borderline-resectable diseases, potentially obscuring the true treatment effect for patients with resectable tumors [52]. Identifying patients who will derive the most benefit from NAT remains a challenge, hindering the optimization of treatment efficacy [53].

Relatively few phase III trials have specifically focused on perioperative therapies for resectable PDAC and concerns about delaying surgery due to potential disease progression have hampered enrollment [9]. Table 2 summarizes the main phase III trials evaluating neoadjuvant therapy in resectable PDAC.

### 5.5. Borderline-Resectable Pancreatic Cancer

The role of neoadjuvant therapy in borderline-resectable disease is promising. In fact, studies suggest it may improve OS without necessarily increasing resection rates [9].

Recent clinical trials have shed light on the efficacy of various treatment regimens and highlighted ongoing areas of investigation for improving outcomes in patients with Borderline Resectable PDAC (BR-PDAC) [52,55,56,57,58].

The ESPAC5 trial, a multicenter, randomized, phase 2 clinical trial, provides the first line of evidence suggesting that short-course neoadjuvant chemotherapy may be more effective than immediate surgery followed by adjuvant therapy for patients with borderline resectable pancreatic cancer [55]. Notably, this study favored chemotherapy over chemoradiotherapy, suggesting radiation may not offer additional benefits in the neoadjuvant setting [55]. Patients were randomly assigned to one of four groups: immediate surgery, neoadjuvant gemcitabine + capecitabine, involving a short course of chemotherapy before surgery; neoadjuvant therapy with FOLFIRINOX, involving a short course of a more intensive chemotherapy regimen before surgery; neoadjuvant chemoradiotherapy, combined short-course chemotherapy with radiation therapy before surgery [55]. The study revealed that short-course neoadjuvant therapy (using any of three methods) was more effective in improving one-year disease-free survival rates compared to immediate surgery (59% vs. 39%). Among the options, neoadjuvant chemotherapy combining gemcitabine with capecitabine or FOLFIRINOX demonstrated the greatest survival benefit. However, further rigorous trials are needed to confirm these findings and refine treatment approaches [55].

The PREOPANC-1 trial investigated the efficacy of neoadjuvant therapy in patients with resectable or borderline-resectable pancreatic tumors. A total of 246 patients were randomized to either upfront surgery followed by adjuvant gemcitabine or neoadjuvant gemcitabine-based chemoradiotherapy (CRT) followed by surgery and adjuvant gemcitabine. The primary endpoint, median overall survival (OS), did not show a statistically significant difference between the two groups (16.0 months with pre-operative CRT vs. 14.3 months with upfront surgery), as reported previously [52]. An unplanned analysis with a longer follow-up period revealed a trend towards improved median OS with neoadjuvant CRT (15.7 months vs. 14.3 months) and a more substantial difference in 5-year OS rates (20.5% vs. 6.5%, respectively), as previously mentioned [21]. A pre-specified subgroup analysis focusing on patients with borderline-resectable disease demonstrated a significant improvement in median OS with neoadjuvant CRT compared to upfront surgery (17.6 months vs. 13.2 months; hazard ratio [HR] 0.62, 95% CI 0.40–0.95; *p* = 0.029) [9].

A021501, a phase II randomized trial conducted by the National Cancer Institute National Clinical Trials Network, was designed to assess the efficacy of two neoadjuvant treatment regimens in patients with borderline resectable PDAC. Arm A employed systemic chemotherapy alone with eight cycles of mFOLFIRINOX, while Arm B incorporated sequential hypofractionated radiotherapy with seven cycles of mFOLFIRINOX [56]. The primary results of A021501 were the following: 18-month OS rate was higher in Arm A (mFOLFIRINOX only) at 67.9% compared to 47.3% in Arm B (mFOLFIRINOX + Radiotherapy), patients who underwent surgery after treatment had a significantly higher 18-month OS rate in both arms (over 90%), Arm B had a higher rate of side effects compared to Arm A [56]. The study suggested that pre-operative mFOLFIRINOX chemotherapy alone might be more effective than mFOLFIRINOX combined with hypofractionated radiotherapy for borderline resectable pancreatic adenocarcinoma, with potentially fewer side effects [56].

Eshmuminov et al. (2023) reported that FOLFIRINOX demonstrated a survival benefit in patients who ultimately did not undergo surgery for BR-PDAC or locally advanced pancreatic cancer (LAPC) compared to gemcitabine-based regimens. However, for patients who did undergo surgery, survival outcomes were similar between the two regimens. This suggests FOLFIRINOX may be preferable for patients with good performance status, while gemcitabine remains a reasonable alternative for those with lower tolerance or those expected to undergo resection [57].

The NUPAT-01 trial, a phase II study, investigated the feasibility and effectiveness of neoadjuvant chemotherapy for patients with borderline-resectable pancreatic cancer who were enrolled in two groups: FOLFIRINOX and GEM/nab-PTX, the latter combining gemcitabine with nab-paclitaxel. A total of 51 patients participated in the study; the majority (84.3%) of patients were able to undergo surgery after neoadjuvant therapy, and R0 resection was achieved in 67.4% of patients [58]. This study suggests that using FOLFIRINOX or GEM/nab-PTX as neoadjuvant therapy is feasible and well-tolerated in patients with borderline-resectable pancreatic cancer. Additionally, a significant proportion of patients achieved complete tumor removal after surgery. However, further research with longer follow-up is needed to assess the impact of these regimens on overall survival [58].

### 5.6. Locally Advanced, Unresectable

The potential for surgical resection of initially unresectable pancreatic cancer following induction therapy represents a significant development in the field, albeit still debated for certain patient populations [9]. NCCN guidelines recommend a 4–6 month course of induction combination chemotherapy followed by either CRT or SBRT for selected patients without systemic metastases. Following this, surgical resection should be considered if feasible, potentially followed by adjuvant chemotherapy if clinically indicated [9].

For 15 years, gemcitabine held the dominant position as first-line therapy for metastatic PDAC in patients with normal bilirubin and good performance status; however, its effectiveness as a single agent remains modest, even with good tolerability, prompting investigation of combination regimens [59]. De La Fouchardière et al. explored the addition of paclitaxel to gemcitabine for metastatic pancreatic cancer previously treated with FOLFIRINOX in a randomized, phase III PRODIGE 65-UCGI 36-GEMPAX UNICANCER clinical trial [59]. In this study comparing the efficacy of gemcitabine with paclitaxel (Arm A) against gemcitabine alone (Arm B) in treating metastatic pancreatic ductal adenocarcinoma, both treatments showed similar overall survival rates with median survival times of 6.4 months and 5.9 months respectively. However, Arm A demonstrated benefits in progression-free survival and objective response rate, suggesting it might be more suitable for patients with aggressive disease who can handle increased side effects from combination therapy [59].

The NEOLAP-AIO-PAK-0113 trial investigated the effectiveness of two treatment regimens for locally advanced pancreatic cancer: Arm A with a combination of nab-paclitaxel (Abraxane) and gemcitabine, Arm B with a combination of nab-paclitaxel and gemcitabine followed by FOLFIRINOX. The researchers compared these two approaches to see if adding FOLFIRINOX after the initial nab-paclitaxel and gemcitabine combination (Arm B) would lead to better outcomes. The primary endpoint was not overall survival but the rate of patients who underwent complete surgical removal of the tumor (R0 resection rate). There was no significant difference in R0 resection rates between the two arms. The study suggests that nab-paclitaxel plus gemcitabine (Arm A) might be as effective and safe as the more complex regimen of nab-paclitaxel, gemcitabine followed by FOLFIRINOX (Arm B) for treating locally advanced pancreatic cancer [60].

A further analysis led by Guggenberger used computed tomography (CT) scans to predict surgical outcomes in patients with locally advanced pancreatic cancer undergoing multiagent induction chemotherapy. By analyzing both baseline and post-chemotherapy CT scans for tumor size, density, and vascular involvement, the study sought to identify correlations between these imaging features and the likelihood of achieving a successful surgical resection (R0 resection). The findings aimed to establish CT scans as a useful non-invasive tool for predicting surgical resectability, which could enhance treatment planning and potentially improve patient outcomes [61].

The phase III CONKO-007 trial evaluated the potential benefit of adding radiotherapy to induction chemotherapy for patients with pancreatic cancer [62]. A total of 495 patients received initial chemotherapy, consisting of either six cycles of FOLFIRINOX or three cycles of gemcitabine [63]. Following induction chemotherapy, the 336 patients who did not show disease progression were randomized into two groups: the experimental arm that received gemcitabine combined with radiotherapy and the control arm, which continued with systemic therapy for an additional 3 months. After re-evaluation, patients were assigned to either surgical exploration (if deemed technically resectable) or continued systemic treatment. Despite similar median overall survival times of 15 months in both groups, the addition of radiotherapy improved the complete pathological response and increased the proportion of patients achieving complete tumor removal (R0 resection) with negative circumferential margins. Importantly, those achieving R0 resection showed markedly better long-term survival. However, the change did not significantly affect the primary study endpoint, the R0 resection rate. The CONKO-007 trial suggests that adding radiotherapy to induction chemotherapy may improve the chances of achieving complete tumor removal with negative margins (R0 resection) but did not translate into a clear overall survival benefit. However, patients who achieved R0 resection had a significantly improved prognosis [9,63].

## 6. Neoadjuvant versus Adjuvant Therapy

Patients in neoadjuvant trials for pancreatic cancer often encompass a broader range of initial diagnoses, including those with potentially resectable or borderline resectable tumors identified through imaging. This can include patients with less favorable prognoses due to disease progression or unforeseen complications that may prevent surgery. Conversely, adjuvant trials involve patients who have successfully undergone surgery and demonstrate good post-operative recovery without early signs of recurrence, ensuring they are fit for chemotherapy. These stringent criteria inherently select patients with a more favorable prognosis [21]. About 20% of patients initially deemed resectable are later found unsuitable for surgery, revealing the limitations of pre-operative assessments. Furthermore, post-operative recovery poses another barrier, with only about half of the patients being fit for subsequent adjuvant chemotherapy. Many adjuvant trials also require additional criteria, like post-operative CT scans showing no recurrence and specific levels of the CA 19–9 tumor marker. Such restrictions may lead to inflated survival estimates in adjuvant trials compared to neoadjuvant trials, which include a wider spectrum of patients. Recognizing these biases is key for accurately interpreting trial data and assessing the real-world benefits of neoadjuvant therapy [21].

Finally, the current standard of care, per NCCN guidelines, remains adjuvant therapy (post-surgical) [9], but neoadjuvant therapy holds considerable promise for improving PDAC treatment outcomes. Further research is critical to optimize treatment regimens and definitively demonstrate the survival benefits of this approach [64]. High-quality evidence from randomized controlled trials and, therefore, robust data will be crucial for establishing neoadjuvant therapy as a standard treatment option for PDAC [65].

## 7. Challenges in Neoadjuvant Therapy and Future Directions

For four decades, the standard approach to PDAC has been upfront surgery, often guided solely by radiological diagnosis. However, neoadjuvant therapy is emerging as a potentially transformative strategy [9].

While NAT offers promising benefits, it also presents challenges, including potential side effects and the risk of delayed surgery, which could allow cancer progression in some cases. Careful patient selection and risk assessment are crucial for optimizing NAT outcomes in PDAC [51].

Several limitations and considerations are important when interpreting the current body of evidence on neoadjuvant therapy for pancreatic cancer. Firstly, the majority of trials conducted to date are phase 2 studies, which inherently limits their generalizability [51]. Larger, well-powered trials are necessary to draw definitive conclusions about the long-term efficacy and safety of these approaches. Moreover, challenges exist in implementing full-dose neoadjuvant FOLFIRINOX regimens, potentially impacting patient compliance and treatment-related toxicities [21]. Additionally, pre-randomization histological confirmation of pancreatic ductal adenocarcinoma is crucial to avoid including patients with other diagnoses that can skew survival data [21]. The real-world applicability of these findings needs to be carefully considered. Labori et al. acknowledge that their inclusion criteria might not reflect real-world practice, as histological confirmation was not mandatory before randomization [51]. Furthermore, limitations exist in using outdated adjuvant regimens like gemcitabine monotherapy when more effective options are available [21].

Pre-operative diagnosis also presents challenges. Versteijne et al. and Labori et al. highlight a notable number of patients ultimately diagnosed with non-pancreatic ductal adenocarcinoma [21,51]. Versteijne et al. emphasize the need for better dissemination of radiologic resectability criteria and potentially real-time central radiology review to ensure a more homogenous study population. Generalizability concerns exist for studies using S-1-based neoadjuvant therapy due to potential pharmacokinetic and pharmacodynamic differences between Western and East Asian populations [55]. Finally, both Ghaneh et al. and Huan et al. acknowledge the limitations of short follow-up periods in definitively assessing long-term patient outcomes, particularly overall survival [55,66].

Several key considerations guide future research directions in neoadjuvant therapy (NAT) for pancreatic cancer. Head-to-head comparisons of different treatment regimens are valuable for informing clinical practice, although existing trials were not designed for this purpose [51]. Establishing standardized and objective criteria for defining resectability in trials is crucial for accurate comparisons and generalizability of results [21].

Moreover, real-time central radiology review during neoadjuvant therapy trials can help ensure patient eligibility and homogeneity of the study population [21]. Future trials should incorporate the latest and most effective adjuvant chemotherapy regimens to accurately assess the overall efficacy of neoadjuvant treatment strategies, and careful consideration is needed to improve the generalizability of findings across populations [21,66]. Studies using S-1-based regimens, for example, require further investigation due to potential pharmacokinetic and pharmacodynamic differences [55]. Long-term follow-up data from ongoing trials is essential for definitively assessing the impact of NAT on overall survival [21] and implementing robust quality control measures is necessary to ensure accurate assessment of resectability and minimize variability in treatment decisions across different institutions [53].

A more comprehensive analysis must include immunotherapy and gene therapy as emerging therapeutic strategies in the treatment of PDAC, encompassing a diverse array of approaches, which include immune checkpoint inhibitors (ICIs), cancer vaccines, adoptive cell therapy (ACT), oncolytic viruses, and matrix-depleting therapies [67].

CAR-T cell therapy is a subset of adoptive cell therapy (ACT) and has appeared as a transformative and promising approach in the management of both malignant and non-malignant diseases [68]. These engineered T cells express synthetic transmembrane receptors (CARs) that enable them to recognize and eliminate target cells expressing specific surface antigens [68]. CAR-modified immune effector cells, encompassing T cells, natural killer (NK) cells, and macrophages, mediate potent cytotoxic antitumor effects through diverse mechanisms, including the perforin-granzyme pathway, the Fas–Fas ligand (FasL) pathway and the secretion of pro-inflammatory cytokines [68]. This specific technique entails a multi-step manufacturing process beginning with the apheresis of a patient’s T lymphocytes, followed by their in vitro activation, genetic modification to incorporate the CAR transgene and subsequent ex vivo expansion; finally, the manufactured CAR-T cells are reinfused back into the patient’s circulation through intravenous administration [68]. The extensive review of the literature done by Czaplicka et al. (2024) identified a limited number of completed clinical trials investigating CAR-T therapy for pancreatic cancer with published data of Phase I studies exploring targets such as MSLN, EGFR, HER2, CLDN18.2 and have revealed both promise and significant challenges in their therapeutic application [68]. Safety assessments indicated generally acceptable tolerability with manageable and reversible adverse effects, and partial responses or stable disease were observed in a limited patient subset, with a substantial proportion exhibiting no response [68]. Despite these initial advancements, the successful implementation of efficacious CAR T-cell therapies for PDAC remains an ongoing pursuit, and definitive success is not yet assured [68]. Certain studies reported poor infiltration of CAR T-cells into the tumor microenvironment, while others documented limited persistence of these cells within the patients [68]. The intricate biological nature of pancreatic cancer necessitates comprehensive and systematic investigations into the molecular mechanisms responsible for the observed shortcomings of currently evaluated treatments; such knowledge is paramount for refining and optimizing the design of next-generation therapies [68]. The field of innovative immunotherapy has also yielded significant progress in pancreatic cancer research, particularly in the identification of promising candidate biomarkers such as myeloid-derived suppressor cells (MDSCs), bone marrow-derived cells with immunosuppressive capabilities; arginine, a critical player in the metabolic landscape of diverse malignant tumors; indole-3-acetic acid (IAA), a tryptophan metabolite generated by two specific gut bacterial strains [67]. All of these biomarkers hold the potential to improve diagnostic and therapeutic precision; specifically, they may enable earlier disease detection, facilitate more accurate assessment of treatment responses, and contribute to improved prognostic prediction [67].

The incorporation of biomarkers into future clinical trials represents a particularly interesting avenue for identifying patients with the greatest likelihood of benefiting from neoadjuvant therapy. This approach has the potential to pave the way for the development of personalized treatment strategies for pancreatic cancer [51].

## 8. Conclusions

This review highlights the evolving landscape of pancreatic cancer treatment, with NAT emerging as a cornerstone strategy. Continued research focused on overcoming chemoresistance and refining response assessment will further improve the efficacy of NAT, ultimately leading to better clinical outcomes and enhanced survival for patients with this malignancy.

## Figures and Tables

**Table 1:** Clinical trials between 1997 and 2018.

Year	Study	Treatment Group	Overall Survival	Survival Rate	Notable Adverse Events	Results
1997 [15,33]	First study on PDAC	Gemcitabine vs. 5-FU	5.65 months vs. 4.41 months	18% vs. 2%	Less toxicity in gemcitabine.	Gemcitabine became the standard of care
2011 [15,16,19,34]	ACCORD11/PRODIGE4	FOLFIRINOX vs. gemcitabine	11.1 months vs. 6.8 months	Degradation of quality of life: 31% vs. 66%	Diarrhea, neuropathy (more in FOLFIRINOX)	Improved and delayed QoL impairment made FOLFIRINOX the preferred strategy
2013 [15,16,35]	MPACT	Nab-paclitaxel + gemcitabine vs. gemcitabine monotherapy	8.5 months vs. 6.7 months	35% vs. 22%	Neutropenia, fatigue, neuropathy (in the nab-paclitaxel-gemcitabine group)	The combination improved overall survival and response rate but more side effects
2016 [15,16,36,37,38]	NAPOLI-1	Nanoliposomal irinotecan + 5-FU/FA, 5 FU/FA, Nanoliposomal monotherapy	6.1, 4.2, 4.9 months	N/A	Neutropenia, diarrhea, vomiting, fatigue (in the nanoliposomal irinotecan/5-FU and folinic acid combination)	Survival benefits of nal-IRI+5-FU/LV versus 5-FU/LV
2017 [39]	ESPAC4	Gemcitabine + capecitabine vs. gemcitabine	28.0 months vs. 25.5 months	N/A	N/A	The adjuvant combination is a better standard of care
2018 [40]	PRODIGE24	Modified FOLFIRINOX vs. gemcitabine	54.4 months vs. 35.0	Longer with mFOLFIRINOX	Higher toxicity in mFOLFIRINOX	Longer survival with FOLFIRINOX at the expense of more toxic effects

**Table 2:** Phase III clinical trials evaluating neoadjuvant therapy in resectable PDAC.

Trial Name	Treatment Approach	Primary Findings	Median OS	Significance
Prep-02/JSAP-05 [9]	Neoadjuvant gemcitabine + S1, surgery, adjuvant S1 vs. Upfront surgery, adjuvant S1	Similar rates of resection, R0 resection, post-operative morbidity, and mortality. The neoadjuvant group had improved OS.	Neoadjuvant: 36.7 months; Upfront: 26.6 months	Neoadjuvant therapy showed improved OS.
Alliance A021806 [53,54]	Perioperative mFOLFIRINOX vs. Surgery followed by adjuvant mFOLFIRINOX	Evaluating efficacy of perioperative mFOLFIRINOX; currently recruiting, outcomes pending.	N/A	Ongoing trial.
PREOPANC [21]	Neoadjuvant gemcitabine-based chemoradiotherapy, surgery, adjuvant gemcitabine vs. Upfront surgery, adjuvant gemcitabine	The neoadjuvant therapy group showed significant long-term survival benefits.	5-year OS improved by 14% in the neoadjuvant group	Significant survival benefit with neoadjuvant therapy.

## Data Availability

No new data were created.
